# Chemical composition and anti-aging effects of standardized herbal chicken essence on D-galactose- induced senescent mice

**DOI:** 10.3389/fnut.2022.989067

**Published:** 2022-09-13

**Authors:** Shu-Jing Wu, Yi-Jou Tung, Ming-Hong Yen, Lean-Teik Ng

**Affiliations:** ^1^Department of Nutritional Health, Chia-Nan University of Pharmacy and Science, Tainan, Taiwan; ^2^Graduate Institute of Natural Products, College of Pharmacy, Kaohsiung Medical University, Kaohsiung, Taiwan; ^3^Department of Agricultural Chemistry, National Taiwan University, Taipei, Taiwan

**Keywords:** chicken essence, anti-aging, D-galactose, anti-inflammation, SIRT1

## Abstract

This study aimed to examine the chemical and anti-aging properties of chicken essence (CE) prepared with *Sesamum indicum, Angelica acutiloba*, and *Zingiber officinale* (HCE). HCE was analyzed for nutritional and phytochemical composition, and its anti-aging effects were investigated on the D-galactose (Gal)-induced aging mice. Results showed that HCE possessed significantly higher calories and contents of valine and total phenols than CE; it also contained significant amounts of ferulic acid, sesamin, and sesamolin. HCE significantly decreased MDA and NO levels in serum and liver and increased liver GSH levels in the D-Gal-induced mice. HCE greatly enhanced SOD and CAT activities in serum and liver, and liver GPx activity, as well as upregulating SIRT1 expression and downregulating TNF-α, IL-1β, IL-6, iNOS, Cox-2, and MCP-1 expression in liver tissues. This study demonstrates that HCE was effective in suppressing the aging process through enhancing antioxidant and anti-inflammatory activities and modulating the aging-related gene expression.

## Introduction

Aging has increasingly become an important public issue in many developed countries. It has been reported to associate with an increased risk of cardiovascular disease, cancer, cognitive impairment, and so on (1). Reactive oxygen species (ROS) driven by oxidative stress and increased inflammatory responses are believed to play an important role in the senescence process (2). D-Galactose (D-Gal; a reducing sugar) is widely used to induce oxidative stress *in vivo* to mimic the natural aging in mice (3, 4). As aging progress, the function of antioxidant defense systems, including superoxide dismutase (SOD), catalase (CAT), and glutathione peroxidase (GPx) deteriorate in efficiency in preventing ROS-induced biological damage (5). Changes in malondialdehyde (MDA, an important marker of oxidative stress *in vivo*) levels in serum or tissues indicate the occurrence of lipid peroxidation, tissue damage, and impairment of the antioxidant defense system (6).

Inflammatory mediators such as TNF-α, IL-1β, IL-6, IL-8, MCP-1, iNOS, COX-2, and adhesion molecules have been reported to increase in advanced age (7–9). The balance between the promoters and inhibitors of immuno-inflammatory responses has been shown to relate to healthy aging and longevity. Monocyte chemoattractant protein-1 (MCP-1) and Sirtuin 1 (SIRT1; silent information regulator T1) are proteins reported to associate with mammalian aging. Circulating levels of MCP-1 are considered a promising biomarker for measuring biological age in mice and human frailty (10). SIRT1 can extend lifespan, delay aging, and prevent aging-related diseases (11), and its level was found to decrease in the liver with age (12). SIRT1 deficiency was reported to promote the expression of aging-related genes (13).

In Asia, chicken essence (CE) has been widely used for anti-stress and anti-fatigue, as well as for enhancing physical strength and cognitive performance (14, 15). Studies have shown that CE contains important nutrients such as carnosine, anserine, taurine, trace elements, and indispensable amino acids (16). It is believed that the prescription containing two or more medicinal herbs in CE is therapeutically more effective than only CE alone. *Sesamum indicum* (black sesame), *Angelica acutiloba* (dang gui), and *Zingiber officinale* (ginger) are locally produced medicinal plants, which are often used in the production of delicious dishes and therapeutic medicated diets. *S. indicum* contains lignans such as sesamin, sesamolin, and sesaminol as active ingredients, which have immunomodulatory, anti-cancer, antioxidative, and cholesterol-lowering effects (17). The active ingredients of *A. acutiloba* include phthalides, organic acids, and polysaccharides, which have anti-cancer, antioxidant, nerve protection, and immune enhancement properties (18). *Z. officinale* contains 6-gingerol and 6-shogaol, which have cardiovascular protection, anti-cancer, antioxidant, anti-inflammatory, and hypoglycemic activities (19). However, no study has been reported on CE or related products prepared with these medicinal herbs.

Although the mechanisms underlying the bioactivities of herbal CE could be complicated and may be regulated by the combined actions of many active components, it is worthwhile to study the health benefits of CE prepared with medicinal herbs. CE has always been considered to have an anti-aging effect in traditional Chinese medicine. However, little study has been reported on its impacts on the aging process. In this study, we aimed to investigate the chemical composition and anti-aging effects of herbal chicken essence (HCE) containing *S. indicum, A. acutiloba*, and *Z. officinale*, and its underlying anti-aging molecular mechanisms in the D-Gal-induced aging mice.

## Materials and methods

### Materials

*Sesamum indicum* (black sesame), *Angelica acutiloba* (dang gui), and *Zingiber officinale* (ginger) were obtained from the local markets (Tainan, Taiwan). D-Galactose (D-Gal), Tween-20, amino acid standards (valine, leucine, and isoleucine), and anti-β-actin were purchased from Sigma Chemical Co. (St. Louis, MO. USA). Primary antibodies (anti-TNF-α, anti-IL-1β, anti-IL-6, anti-IL-10, anti-MCP-1, anti-SIRT1, anti-iNOS, and anti-Cox-2) and secondary antibodies (anti-rabbit and anti-mouse) were obtained from Cell Signaling Technology, Inc. (Beverly, MA, USA). Nitric oxide (NO) assay kit was obtained from Promega Corporation (Madison, WI, USA). Superoxide dismutase (SOD), catalase (CAT), glutathione peroxidase (GPx), glutathione (GSH), and malondialdehyde (MDA) assay kits were purchased from Cayman Chemical Company (Ann Arbor, MI, USA). All other chemicals used in this study were of analytical grade.

### Preparation of concentrated chicken essence

The chicken essence (CE) was prepared under good manufacturing practices and hazard analysis (Tsing Shang Co., Ltd., New Taipei City, Taiwan). In brief, 10 kg of black feather native chicken (*Gallus gallus domesticus*) of Taiwan was cooked at high temperature under high pressure with and without medicinal herbs (i.e., 70 g of *S. indicum*, 80 g of *A. sinensis*, and 50 g of *Z. officinale*) at 121°C for 3 h to produce the CE and HCE extracts, respectively. To ensure precise and accurate dosing of animals, the extracts were filtered and lyophilized to obtain the concentrated pastes of CE and HCE, which were collected and stored at −80°C until use.

### Analysis of general nutritional components

The contents of calories, protein, fat, carbohydrates, cholesterol, trans fatty acids, and sodium in samples were analyzed according to the AOAC standard methods. Branched-amino acids such as valine, leucine, and isoleucine were quantified by HPLC analysis (20).

### Analysis of total phenol and bioactive phytochemical contents

In brief, 50 mg of CE and HCE were taken and well-mixed with 1 mL methanol, followed by sonication at room temperature for 30 min. After filtration, the filtrates were collected for chemical analysis.

The total phenol content was determined by the Folin–Ciocalteu method (21), and the values were expressed as grams of gallic acid equivalent (GAE) per kg of CE. The bioactive phytochemical contents were determined by high performance liquid chromatographic system (HPLC; Hitachi Co. Inc., Japan), equipped with a quaternary pump (L-7100), an autosampler (L-7200), a UV-VIS detector (L-7420), and a Mightysil RP-18 GP column (5 μm, 4.6 x 250 mm; Kanto Chemical Co. Inc., Tokyo, Japan). The mobile phase composed of (A) aqueous phosphoric acid (0.1%, v/v) and (B) acetonitrile, with a gradient elution of 0% B at 0 to 3 min, 0 to 20% B at 3 to 15 min, 20–35% B at 15 to 40 min, 35–60% B at 40 to 80 min, 60% B at 80 to 90 min, 100% B at 90–100 min. The flow rate was 1.0 mL/min, the injection volume of the sample was 20 μL, and the detection wavelength was 270 nm. The chromatographic data were recorded and processed with D-7000 Muti-HSM Manager software. The contents of bioactive phytochemicals were quantified using calibration curves of their respective standards (i.e., ferulic acid, 6-gingerol, sesamin, sesamolin, and 6-shogaol).

### Animals and experiment design

C57BL/6J strain mice (6 weeks old) were purchased from BioLASCO Taiwan (Yi-Lan County, Taiwan). They were given a standard laboratory diet (No. 5001; PMI Nutrition International, Brentwood, MO, USA) and distilled water *ad libitum*, and were maintained at 12 h light/12 h dark cycle, at a temperature of 22 ± 2°C and a humidity of 50% to 60%. Ethical approval for the use of animals was obtained from the Institutional Animal Ethical Committee of Chia-Nan University of Pharmacy and Science (Tainan, Taiwan), with the protocol approval number CN-IACUC-103002R. The study was carried out in accordance with the Guide for the Care and Use of Laboratory Animals (National Institutes of Health, MD, USA, 1996).

After a week of acclimation, the mice were randomly divided into five groups with 10 mice per group, namely: (1) a normal (Control) group, (2) an aging model (Model; D-Gal only) group, (3) low HCE (D-Gal+12.5 mg/kg/day HCE; LHCE) group, (4) medium HCE (D-Gal+25 mg/kg/day HCE; MHCE) group, and (5) high HCE (D-Gal+50 mg/kg/day HCE; HHCE) group. To establish a natural aging model mouse, D-Gal (100 mg/kg/day) was injected subcutaneously into the mice of the model and treatment groups once a day for 8 weeks, while mice in the normal group received equal amounts of physiological saline once daily instead of D-Gal. After subcutaneous injection of D-Gal for an hour, mice in normal and model groups were intragastrically (IG) administered with distilled water (vehicle), and mice in treatment groups were IG given their respective doses of HCE. The dosing volume was 10 mL/kg body weight.

Twenty-four hours after the last administration, mice were euthanized with 95% CO_2_ asphyxiation per ethical guidelines. Blood was collected through cardiac puncture and centrifuged at 1,500 ×g at 4°C for 15 min to obtain serum, which was collected and stored at −80°C until analysis. The liver tissues were homogenized in icy lysis buffer. After centrifugation at 10,000 ×g at 4°C for 10 min, the supernatants were collected for biochemical and Western blot analyses.

### Observation of general appearance, body weight, and relative organ weight

The general appearance of the mice was observed weekly during the entire experiment, including mental condition, hair, urine, and stool. Food and water consumption were monitored daily, and the body weight of the animals was measured every 3 days. The dose received by each animal was calculated based on the individual animal's body weight and adjusted according to the subsequent changes in body weight.

### Measurements of antioxidants, malondialdehyde, and nitric oxide

The protein concentrations were measured by the bicinchoninic acid method using bovine serum albumin as a standard. The activities of SOD, CAT, and GPx, as well as the levels of GSH, MDA, and NO in serum and liver, were determined according to the instructions provided by the assay kit suppliers.

### Western blotting analysis

The Western blot analysis was employed to detect the protein expression levels of TNF-α, IL-1β, IL-6, iNOS, COX-2, MCP-1, and SIRT1 in liver tissues. In brief, 50 μg/lane of lysate protein was taken and separated by electrophoresis on sodium dodecyl sulfate (SDS)-polyacrylamide gels, followed by transfer to polyvinylidene difluoride membranes. The membrane was then incubated in a blocking solution containing bovine serum albumin (1%) and Tween-20 (0.1% v/v) in phosphate-buffered saline at room temperature for 1 h with the appropriate primary antibodies. After washing with TBS-T (Tris buffered saline with Tween-20), the membranes were incubated with appropriate secondary antibodies at room temperature for 2 h, followed by washing with TBS-T. The immune-reactive bands were detected with HRP staining and then developed using an enhanced ChemiImager 5500 chemiluminescence system (Alpha Innotech Corporation, Miami, FL, USA). Here, β-Actin served as a loading control.

### Statistical analysis

Data are expressed as mean±SD. Differences between CE- and HCE-treated groups were analyzed by Student's *t*-test. A one-way analysis of variance (ANOVA) and *post-hoc* Duncan's multiple range test were used to evaluate the difference in means between treatments. A *P* < 0.05 was considered statistically significant.

## Results

### Extraction yield and nutritional composition

The yields of concentrated chicken essence (CE) and herbal chicken essence (HCE) were 17.68% and 19.80%, respectively. The caloric and sodium contents of HCE were significantly higher than CE (Table 1). The addition of medicinal herbs has increased the sugar and protein contents of CE, which may have led to a higher calorie content in HCE.

**Table 1:** Contents of nutritional components and total phenols in different types of chicken essence.

	**CE**	**HCE**
Gross energy (Kcal)	20.40 ± 0.88	24.60 ± 0.96*
Protein (%)	5.10 ± 0.22	5.70 ± 0.19*
Lipid (%)	ND	ND
CHO (%)	ND	0.20 ± 0.08
Ash (%)	0.73 ± 0.08	0.80 ± 0.01*
H_2_O (%)	94.17 ± 0.25	93.30 ± 0.15
Cholesterols (%)	ND	ND
Trans-fatty acid (%)	ND	ND
Na (mg)	39.02 ± 0.84	47.66 ± 1.02*
Ca (ppm)	ND	2.00 ± 0.01
Valine	180.9 ± 1.5	189.4 ± 3.7*
Leucine	250.8 ± 4.6	248.9 ± 0.4
Isoleucine	118.4 ± 1.9	119.8 ± 0.3
Total phenols	511.1 ± 38.9	878.8 ± 75.5*

Values are means ± SD of three independent experiments. CE: Normal chicken essence. HCE, Herbal chicken essence; ND, not detected. The asterisks indicate a significant difference between CE and HCE at P < 0.01 as analyzed by Student's t-test.

### Contents of branched-chain amino acids, total phenols, and bioactive phytochemical constituents

**Figure 1:**
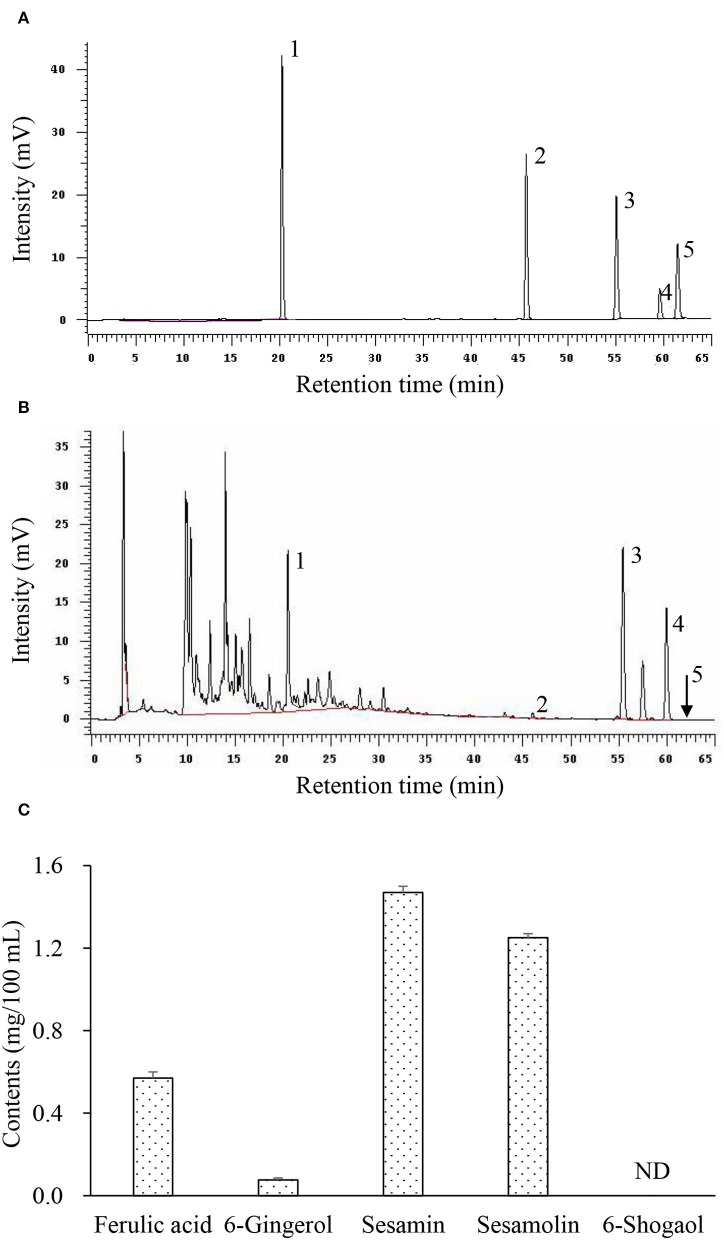
Representative chromatograms of bioactive phytochemicals in herbal chicken essence (HCE) as determined by HPLC. **(A)** Standard references; **(B)** Herbal chicken essence; **(C)** Contents of bioactive phytochemicals. 1, Ferulic acid; 2, 6-Gingerol; 3, Sesamin; 4, Sesamolin; 5, 6-Shogaol; ND, Not detected. Values are expressed as means ± SD (*n* = 3).

Table 1 shows the total branched-chain amino acid content (about 558 mg/100 ml) in HCE and CE (about 550 mg/100 ml). Results showed that the valine content (189.4 mg/100 ml) in HCE was significantly higher than in CE (180.9 mg/100), while the difference in leucine and isoleucine contents was not statistically significant. In addition, the total phenol content (878.8 mg/100 ml) in HCE was also significantly higher than CE (511.1 mg/100 ml); this suggests that the increased release of phenolic compounds from the medicinal herbs into the dripping chicken extract, and thus led to a higher content of these compounds in HCE. Besides 6-shogaol, HCE was shown to contain various bioactive phytochemicals, namely ferulic acid, 6-gingerol, sesamin, and sesamolin (Figure 1), but these compounds were not present in CE as it contained only the essence of chicken. Furthermore, the chickens used to prepare the essence of this study were also never fed on a diet containing *S. indicum, A. acutiloba*, and *Z. officinale*, neither they were added to the CE during preparation.

### General appearance, body weight gain, and relative organ weights

Based on visual observations, rats receiving HCE were noted to be more active and had the sign of a healthy appearance. However, mice of the model group (animals receiving D-Gal injection only) showed obvious symptoms of aging, such as slow movement, a lag in response, listlessness, and withered and lackluster fur, indicating that the D-Gal-induced aging model was successfully established.

Among the animals receiving different doses of HCE, there was no pathological appearance and abnormal behavioral activities during the 8-week experimental period. Body weight change was not significantly different between mice of the control group and those receiving medium (MHCE) and high (HHCE) doses of HCE (Table 2). There were no animals that died due to the D-Gal and HCE treatments. Although there was slightly lackluster fur and a slow reaction for mice receiving low-dose HCE, the senescence phenomenon was moderated as compared to those in the model group. These observations suggest that the different dosages of HCE did not affect the normal body metabolism of animals and are not harmful to their growth. Besides D-Gal alone treated animals, the relative liver weight among control and HCE-treated animals was not statistically significant (Table 2), indicating that HCE on prolonged intake did not affect the normal functions of organs.

**Table 2:** Effects of different doses of herbal chicken essence (HCE) on body weight gain, liver weight, and relative liver weight of D-galactose-induced aging mice.

**Groups**	**Body weight gain (g)**	**Liver weight (g)**	**Relative liver weight (%)**
Control	5.76 ± 0.56^a^	1.47 ± 0.12^bc^	5.46 ± 0.22^bc^
Model	4.17 ± 0.80^c^	1.62 ± 0.09^a^	5.86 ± 0.24^a^
LHCE	4.64 ± 0.14^bc^	1.50 ± 0.08^bc^	5.59 ± 0.26^ab^
MHCE	5.27 ± 0.72^ab^	1.42 ± 0.04^c^	5.25 ± 0.22^bc^
HHCE	5.57 ± 0.56^a^	1.49 ± 0.07^bc^	5.02 ± 0.45^c^

Data are expressed as means ± SD (n = 6). Control, normal group; Model, D-Gal-induced group; LHEC, D-Gal+12.5 mg/kg/day, low HCE group; MHEC, D-Gal+25 mg/kg/day, medium HCE group; HHEC, D-Gal+50 mg/kg/day, high HCE group. Means with different letters in the same column were significantly different at P < 0.05 as analyzed by Duncan's multiple range tests.

### Contents of nitric oxide, malondialdehyde, and glutathione in serum and liver tissues

Compared with the control group, the levels of NO and MDA in serum and liver tissues of the D-Gal treated group were significantly higher, while a significant decrease in liver GSH content was noted (Table 3). In contrast, co-administration of D-Gal with HCE significantly reduced the levels of NO and MDA in serum and liver tissues and increased the liver GSH level. These results suggest that HCE might be involved in preventing D-Gal-induced aging *via* reducing the oxidative stress level in the body of mice.

**Table 3:** Effects of different doses of herbal chicken essence (HCE) on nitric oxide (NO), GSH, and MDA levels and activities of SOD, CAT, and GPx in the serum and liver tissues of D-galactose-induced aging mice.

**Group**	**Serum**			**Liver**		
	**NO (μM)**	**MDA (μM)**	**GSH (μM)**	**NO (μM)**	**MDA (μM)**	**GSH (μM)**
Control	133.3 ± 17.4^b^	10.1 ± 3.6^b^	72.1 ± 5.8^a^	322.2 ± 25.5^b^	21.9 ± 8.4^ab^	292.2 ± 2.1^a^
Model	159.4 ± 13.2^a^	15.6 ± 3.7^a^	68.0 ± 1.7^a^	429.3 ± 34.3^a^	30.2 ± 8.1^a^	259.4 ± 9.7^c^
LHCE	93.6 ± 10.4^c^	8.75 ± 1.78^b^	67.2 ± 0.6^a^	241.7 ± 60.0^c^	29.1 ± 8.4^a^	273.9 ± 14.9^b^
MHCE	60.2 ± 13.7^d^	8.06 ± 2.46^b^	69.8 ± 7.3^a^	190.3 ± 41.2^cd^	28.5 ± 4.0^a^	287.3 ± 15.5^ab^
HHCE	30.9 ± 9.8^e^	5.83 ± 1.52^c^	73.6 ± 6.4^a^	161.2 ± 35.8^d^	13.4 ± 5.8^b^	293.3 ± 2.3^a^
	**SOD**	**CAT**	**GPx**	**SOD**	**CAT**	**GPx**
	**(U/mol)**	**(nmol/min/ml)**	**(nmol/min/ml)**	**(U/mol)**	**(nmol/min/ml)**	**(nmol/min/ml)**
Control	51.2 ± 4.2^ab^	49.7 ± 8.2^a^	118.9 ± 11.6^a^	10.5 ± 0.7^a^	126.9 ± 10.6^bc^	46.3 ± 6.3^bc^
Model	42.3 ± 3.1^c^	30.1 ± 4.8^b^	113.8 ± 11.7^a^	5.64 ± 1.36^b^	114.1 ± 16.1^c^	39.3 ± 9.7^c^
LHCE	47.8 ± 7.9^bc^	39.7 ± 9.5^ab^	117.2 ± 12.5^a^	6.80 ± 1.21^b^	127.4 ± 8.3^bc^	35.4 ± 2.8^c^
MHCE	55.3 ± 7.4^ab^	39.9 ± 9.9^ab^	120.6 ± 11.2^a^	11.1 ± 1.5^a^	134.6 ± 15.7^b^	51.2 ± 7.9^b^
HHCE	58.5 ± 7.9^a^	46.0 ± 9.0^a^	128.2 ± 11.6^a^	11.2 ± 2.1^a^	170.1 ± 19.3^a^	67.1 ± 12.4^a^

Data are expressed as means ± SD (n = 6). Control, normal group; Model, D-Gal-induced group; LHEC, D-Gal+12.5 mg/kg/day, low HCE group; MHEC, D-Gal+25 mg/kg/day, medium HCE group; and HHEC, D-Gal+50 mg/kg/day, high HCE group. Means with different letters in the same column were significantly different at P < 0.05 as analyzed by Duncan's multiple range tests.

### Activities of superoxide dismutase, catalase, and glutathione peroxidase in serum and liver tissues

Compared with the control mice, results showed that mice treated with D-Gal alone significantly reduced SOD and CAT activities in serum and liver tissues, and liver GPx activity; however, serum GPx activity was minimally affected (Table 3). Co-feeding HCE to the D-Gal-induced animals for 8 weeks significantly enhanced the activities of SOD and CAT in serum and liver tissues and GPx activity in the liver, but the serum GPx activity was not greatly affected; the most noticeable improvement in these enzyme activities was noted in the high dose HCE-treated animals. These results suggest that HCE, especially the medium (MHCE) and high (HHCE) doses, can significantly improve SOD, CAT, and GPx activities in serum and liver tissues of the D-Gal-induced aging mice.

### Production of cytokines (TNF-α, IL-1β, and IL-6) in liver tissues

**Figure 2:**
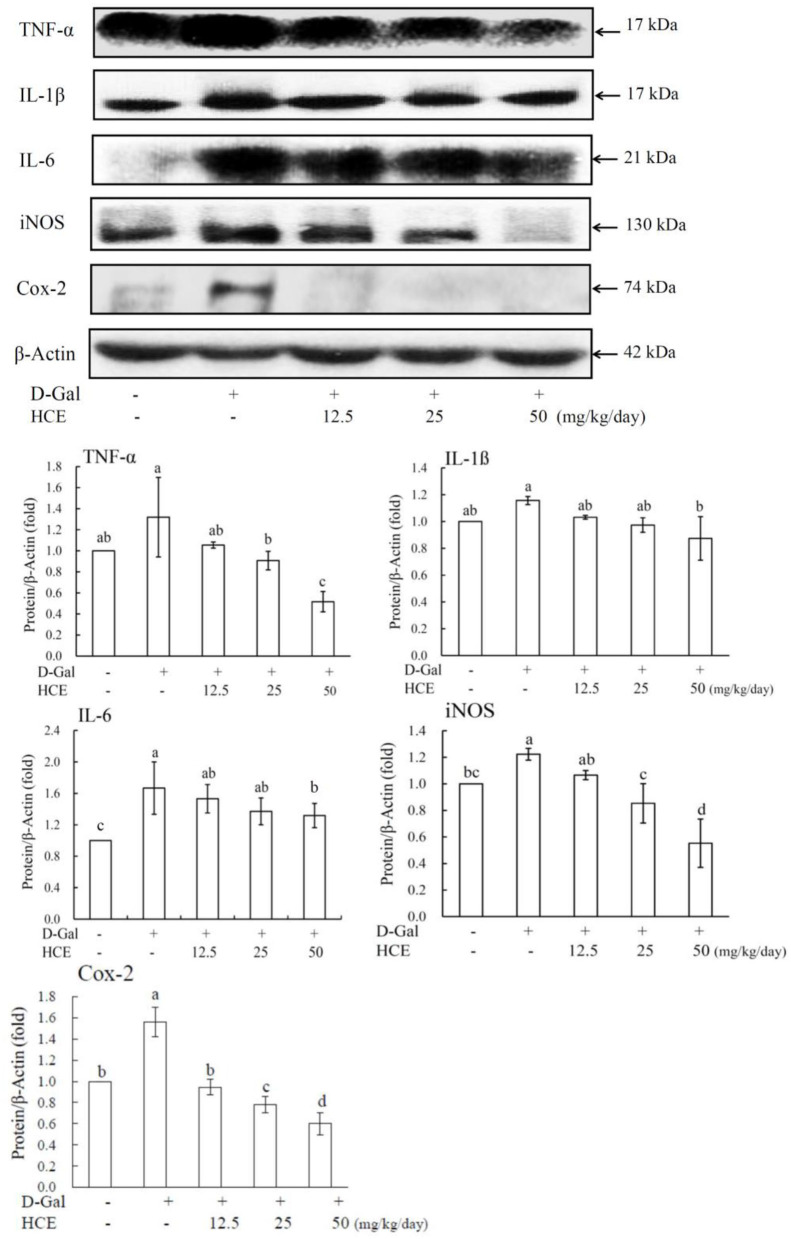
Effects of herbal chicken essence (HCE) on TNF-α, IL-1β, IL-6, iNOS, and Cox-2 production in the liver tissue of D-galactose-induced aging mice. β-Actin was used as a loading control. Values are expressed as means ± SD (*n* = 6). The bar having the same letter was not significantly different at *P* < 0.05 as analyzed by Duncan's multiple range tests.

Results showed that D-Gal alone caused a significantly increased cytokine (TNF-α, IL-1β, and IL-6) release in liver tissues of mice (Figure 2). Administration of HCE significantly reduced the production of these pro-inflammatory cytokines in D-Gal-induced animals, and the high dose of HCE (50.0 mg/kg/day) was shown to have the most effective protection on liver tissues from the inflammatory response. These results demonstrate that HCE was effective in downregulating the secretion of pro-inflammatory cytokines in D-Gal-induced mice, indicating that HCE possesses anti-inflammatory efficacy.

### Expression of iNOS, Cox-2, MCP-1, and SIRT1 proteins in liver tissues

To further understand the delayed aging efficacy of HCE, western blotting was used to analyze the expression of target molecular proteins (iNOS, Cox-2, MCP-1, and SIRT1) in D-Gal-induced mice. Compared to the control group, the results revealed that D-Gal significantly induced inflammation in the liver of mice, as demonstrated by the enhanced expression of iNOS and COX-2 proteins (Figure 2). Compared to animals receiving D-Gal alone, co-treatment of D-Gal plus HCE significantly reduced iNOS and Cox-2 expression, suggesting the effective role of HCE against inflammation in the liver of D-Gal-induced mice.

**Figure 3:**
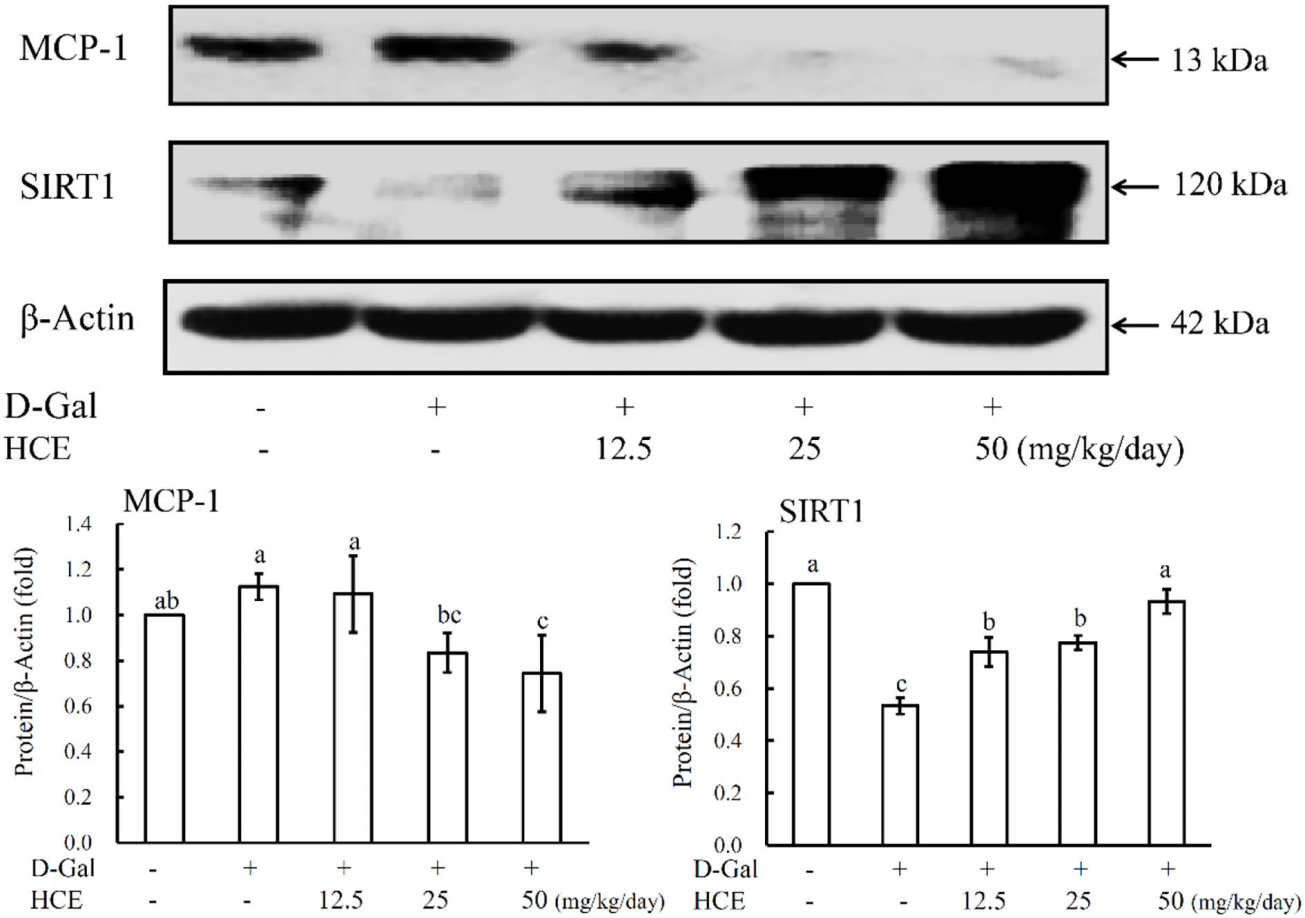
Effects of herbal chicken essence (HCE) on the expression of MCP-1 and SIRT1 in the liver tissue of D-galactose-induced aging mice. β-Actin was used as a loading control. Values are expressed as means ± SD (*n* = 6). The bar having the same letter was not significantly different at *P* < 0.05 as analyzed by Duncan's multiple range tests.

From the results of Figure 3, D-Gal-induced mice alone significantly increased the expression of MCP-1 protein, while significantly reducing the SIRT1 expression in liver tissues. However, co-administration of D-Gal with HCE reduced the expression of MCP-1 protein in liver tissues of mice, and the high dose HCE (50.0 mg/kg/day) treatment exhibited the most significant effect. HCE treatments also significantly reversed the downregulation of SIRT1 expression in the D-Gal-induced animals, and the expression was noted to increase dose-dependently with increasing HCE concentrations. Taken together, in addition to inhibiting iNOS and COX-2 expression, HCE was also effective in suppressing MCP-1 expression and enhancing the expression of SIRT1 protein in the D-Gal-induced mice; these observations further suggest that HCE possesses anti-inflammatory and anti-aging effects.

## Discussion

Human aging has been known to be an irreversible and detrimental process (22), and it is believed to be the result of oxidative stress (2). Numerous studies have revealed that the imbalance between ROS and antioxidant systems in aging may cause progressive loss of tissue and organ function (23). Studies have shown that enzymatic antioxidants (SOD, CAT, and GPx) can protect the liver and kidney from D-Gal-induced oxidative stress in aging by scavenging ROS (4). In this study, D-Gal was injected into mice to induce aging experimentally based on oxidative stress enhancement. At the same time, HCE was orally supplemented to examine its anti-aging protective role against age-associated oxidative stress. Results revealed that administration of D-Gal led to increased oxidative stress, decreased antioxidant enzyme activity, and increased MDA level, which is in line with previous studies (4, 24), indicating that the D-Gal-induced aging model in mice was successfully established. Co-treatment of HCE with D-Gal significantly enhanced the activities of these antioxidant enzymes, and to an extent, higher than those of the model group mice, and also significantly decreased MDA production; this observation indicates that HCE can diminish oxidative stress in the aged mice by enhancing the activities of serum and hepatic antioxidant enzymes (SOD, CAT, and GPx), and reducing the serum MDA level. It is possible that the bioactive compounds, such as ferulic acid and other polyphenolic compounds present in *S. indicum, A. acutiloba*, and *Z. officinale* might potentiate the function of the antioxidant enzyme defense system, hence reducing the oxidative stress *in vivo*.

In this study, one of the main reasons for not including a CE-treated aging group is that chicken essence (CE) is mainly used for anti-stress, anti-fatigue, and for enhancing physical strength (14, 15), and these bioactivities were shown to be related to nutrients such as carnosine, anserine, taurine, and indispensable amino acids. However, no study was reported on CE or related products prepared with popular medicinal herbs known to possess phytonutrients with potent antioxidant and anti-inflammatory activities, and with potential anti-aging effects (25–28). Hence, this study focused only on testing the hypothesis that CE supplemented with medicinal herbs, which are often used in the production of delicious dishes and therapeutic medicated diets, could retard the aging process.

Plant polyphenols have been shown to exert antioxidative effects in animals and humans in clinical studies (29, 30); they act as antioxidants by increasing the activities of SOD, GPx, and CAT (31). D'Antona et al. (32) reported that branched-chain amino acid supplements could prolong the lifespan and provide anti-aging effects in mice through increasing SIRT1 expression, upregulating SOD and GPx activities, and reducing ROS production in middle-aged mice. In another study, resveratrol was shown to prolong the life of yeast and has anti-aging effects *via* the enhanced expression of SIRT1 (33, 34), as well as inhibiting the expression of pro-inflammatory mediators such as iNOS, Cox-2, and NF-κB (35). Ferulic acid has been reported to act as a pleiotropic agent in various age-related diseases for its potent antioxidant and anti-inflammatory properties (3, 36); its treatment significantly reversed the memory impairment in D-Gal-treated mice (3). Besides inhibiting D-Gal-induced oxidative stress *via* increased SOD activity and reduced GSH content, ferulic acid effectively decreased MDA and NO levels. Also, it inhibited inflammation by reducing pro-inflammatory mediator expression. It can also boost the expression of SIRT1, which functions to promote cellular survival and healthy longevity (28); this suggests that ferulic acid has the potential to be used as an anti-aging agent.

Besides ferulic acid, sesamin, sesamolin, and 6-gingerol of HCE may also contribute to the anti-oxidative and anti-inflammatory effects or modulation of anti-aging gene expression. Studies have shown that sesamin and sesamolin can prevent brain damage (37), suppress aging phenotypes (26), and extend lifespan (25); their protective effects on hypoxic neuronal cells are related to the suppression of ROS generation and modulation of mitogen-activated protein kinases. Sesamin possesses potent antioxidant and anti-inflammatory activities (38, 39) and play a role in scavenging free radicals and lipid and glucose metabolism (40). It can also protect the doxorubicin-induced cardiotoxicity through the alleviation of oxidative stress injury and Mn-SOD dysfunction and the activation of SIRT1 (38).

As for 6-gingerol, it was reported to exert potent antioxidant capacity by direct free radical scavenging and triggering of the Nrf2 signaling pathway (41), and is also effective in reducing oxidative stress and inflammation as demonstrated by lowering the levels of MDA, TNF-α, IL-6, IL-1β, iNOS, and C-reactive protein (27, 42). It was found to inhibit lung fibroblast proliferation, reduce inflammation, ensure fibrotic matrix protein deposition via activating SIRT1, and increase the survival rate of mice (43). Other studies showed that 6-gingerol could prolong the lifespan of *Caenorhabditis elegans*, as well as increase thermal and oxidative stress tolerances, antioxidant enzyme activity, expression of heat shock and oxidative stress resistance proteins, and decreased intracellular ROS and lipofuscin accumulation (44). SIRT1 is reported to be the target of 6-gingerol (42). These results indicate that sesamin, sesamolin, and 6-gingerol, present abundantly in HCE, may have played an important role in the anti-aging effects.

Owing to the complexity of the aging process and no universally acceptable single-measurement biomarker of aging is presently known (45), the evidence from several findings, in both animal and human liver specimens, has indicated the presence of specific aging hallmarks in the diseases involving the hepatic compartments; thus, the liver is regarded as an important organ for use in evaluating the aging biomarkers (45–47). D-Gal-induced aging in mice can cause hepatic cell swelling, necrosis, inflammatory cell infiltration, and other pathological changes, leading to liver damage (48). D-Gal also causes oxidative stress damage in the body and secretion of inflammatory cytokines such as IL-1β, IL-6, and TNF-α, as well as resulting in apoptosis and necrosis of hepatocytes (49). Biomarkers of oxidative aging levels in the body are MDA, SOD, GSH, GPx, CAT, and NO; among these markers, aging is closely related to the body's SOD activity and MDA content. The antioxidant enzymes', SOD, GPx, and CAT, activities in aged liver tissue were significantly lower than normal (49). Besides, the reduction of SIRT1 was observed in the livers of old mice, which exhibited impaired body homeostasis and inhibition of liver proliferation (50).

Chronic inflammation plays a key role in the development and progression of many age-related chronic diseases such as atherosclerosis, diabetes, obesity, sarcopenia, and Alzheimer's disease (51). ROS oxidative modification has been reported to induce inflammation in various age-related chronic diseases (52). Senescent cells release proinflammatory cytokines, such as TNF-α, IL-1, IL-6, and IL-8, which cause damage to the surrounding tissues, and are considered highly important in the pathogenesis of inflammaging (8). Several studies have reported that age-related upregulation of the expression of the proinflammatory genes, TNF-α/β, IL-1β, IL-2, IL-6, and IL-8 (7, 51). In this study, inflammatory markers, iNOS, and COX-2, were significantly upregulated in the liver in response to D-Gal-induced oxidative stress. D-Gal also caused a significant increase in the expression of other pro-inflammatory mediators such as NO, TNF-α, IL-1β, and IL-6 in mice. Our finding is consistent with the previous results indicating D-Gal-activated iNOS and COX-2, and increased expression of pro-inflammatory mediators including NO, TNF-α, IL-1β, and IL-6. Conversely, HCE inhibited the activation of iNOS, COX-2, and the release of downstream pro-inflammatory markers. These results further suggest that the phytonutrients of medicinal herbs may have contributed to the anti-inflammatory activity of HCE.

The expression levels of MCP-1 and SIRT1 are closely associated with mammalian aging. Previous studies have demonstrated that plasma levels of MCP-1 correlate with chronologic age in humans (53, 54) and mice (55). SIRT1 has been reported to affect various biological functions, including DNA repair, energy metabolism, tumor suppression, and mitochondrial homeostasis (56). It plays an important role in preventing oxidative stress and inflammation (57). Studies have shown that the downregulation of SIRT1 levels leads to increased oxidative stress and inflammation *in vitro* and *in vivo* (13, 58), which are through the modulation of the SIRT1 downstream pathways, such as NF-κB, PPAR-γ, AMPK, and others. Elevated SIRT1 activity was believed to have beneficial effects on mammal aging and age-associated chronic diseases (56, 59). SIRT1 expression has been reported to decline with age at the transcriptional and translational levels in the brain, liver, skeletal muscle, and white adipose tissues (60). In this study, D-Gal was shown to upregulate MCP-1 expression while downregulating the expression of SIRT1 in liver tissues of mice. However, HCE was effective in suppressing MCP-1 expression and upregulating the expression of SIRT1 as compared with D-Gal alone treated mice. Although we did not experiment by using an inhibitor to block the expression of SIRT1 and to examine if the anti-aging effects of HCE would be abolished, the SIRT1 expression in liver tissues of D-Gal-induced mice was significantly downregulated as compared to the control animals (mice receiving no treatments), while treating the D-Gal-induced aging mice with HCE dose-dependently increased the SIRT1 expression, and its levels were significantly higher than the D-Gal treated mice. These results suggest that HCE possesses bioactive phytonutrients of medicinal herbs, which may provide beneficial anti-aging effects by regulating aging-related gene expression.

The quality of herbal materials is well-known to be affected by factors such as origin, harvesting time, cultivation sites, post-harvesting processing, procedures in extraction and preparation, and others (61). Quality consistency evaluation becomes necessary for quality assurance of finished HCE products. Hence, standardization of herbal materials and procedures is essential for minimizing the difference in effect on anti-aging properties of HCE due to the batch effect. Besides implementing the multivariate data analysis and real-time monitoring of the production process, the establishment of biological and chemical fingerprints of a product manufactured under a standardized procedure was adopted as a reference for controlling the batch-to-batch quality consistency of HCE.

## Conclusion

This study has demonstrated that HCE effectively reduces the aging effects of D-Gal-induced mice. This effect may be mediated, at least in part, by enhancing antioxidant activity, inhibiting pro-inflammatory mediator production, and modulating aging-related gene expression. To the best of our knowledge, this was the first study reporting on the effects of HCE on oxidative stress and aging-associated gene expression in an animal model. As the mechanisms of action of HCE are complicated and may be mediated *via* the combined actions of many functional substances, further long-term investigations should be conducted to substantiate its anti-aging mechanism(s) of action. This study concludes that HCE could be used as a promising dietary supplement for delaying the senescence process.

## Data availability statement

The original contributions presented in this study are included in the article, further inquiries can be directed to corresponding author.

## Ethics statement

The animal study was reviewed and approved by the Institutional Animal Ethical Committee of Chia-Nan University of Pharmacy and Science (Tainan, Taiwan).

## Author contributions

S-JW: conceptualization, methodology development, supervision, and writing the original draft. Y-JT: experimental work and data analyses. M-HY: data analyses. L-TN: data interpretation, critical revision, and editing. All authors contributed to the article and approved the submitted version.

## Funding

This study received funding from the Industrial Development Bureau (Ministry of Economic Affairs of Taiwan) and Pure Day Ltd., Co. (New Taipei City, Taiwan). The funders were not involved in the study design, collection, analysis, interpretation of data, the writing of this article or the decision to submit it for publication.

## Conflict of interest

The authors declare that the research was conducted in the absence of any commercial or financial relationships that could be construed as a potential conflict of interest.

## Publisher's note

All claims expressed in this article are solely those of the authors and do not necessarily represent those of their affiliated organizations, or those of the publisher, the editors and the reviewers. Any product that may be evaluated in this article, or claim that may be made by its manufacturer, is not guaranteed or endorsed by the publisher.
